# Consumer Food Environment Healthiness Score: Development, Validation, and Testing between Different Types of Food Retailers

**DOI:** 10.3390/ijerph18073690

**Published:** 2021-04-01

**Authors:** Camila Aparecida Borges, Kamila Tiemann Gabe, Patricia Constante Jaime

**Affiliations:** Center for Epidemiological Research in Nutrition and Health, Department of Nutrition, Faculty of Public Health–University of Sao Paulo, Av. Dr. Arnaldo, 715-Cerqueira César, Sao Paulo 01246-904, Brazil; ktgabe@usp.br (K.T.G.); constant@usp.br (P.C.J.)

**Keywords:** nutrition consumer environment, food retailer, score, healthiness

## Abstract

The aim of this study was to develop and validate a scoring system, based on AUDITNOVA, to assess the healthiness of the consumer food environment, considering food availability, price, advertising, and placement strategies. Audited data of 650 food retailers were used to develop, validate, and test the consumer food environment healthiness score. To compose the score, the reference was the Dietary Guidelines for the Brazilian Population. The total and subscores were standardized for a scale from 0 to 100. Construct validity was assessed using the Kruskal–Wallis Dunn tests. Cronbach’s alpha coefficients were calculated to determine the consistency of the scores. The median score was 33.7 (p25 = 26.9; p75 = 42.1). The public and private specialized indoor fresh food markets showed the highest medians; otherwise, bakeries and food retailers with the predominant sale of ultra-processed foods showed the lowest. The score was able to satisfactorily classify the extreme food retailer groups by the predominant sale of fresh or minimally processed foods and the predominant sale of ultra-processed foods. The results of Cronbach’s alpha showed excellent internal consistency (α = 0.91). The score helped to provide an overall assessment of consumer food environment healthiness and was able to classify food retailer groups as healthy and unhealthy according to the degree of processing of the available foods.

## 1. Introduction

Access to adequate and healthy food is influenced by socioeconomic, behavioral, and cultural factors which are influenced by the environment [1]. Different theoretical models on food environment have converged in recognizing its complexity, multidimensionality, and potential influence on food choices and practices of individuals and collectivity [2,3,4,5]. In this context, the ecological model of Glanz et al. (2005) stands out [3], proposing that health-related behavior and, in particular, eating practices, are influenced by political, environmental, and individual components. According to this model, the food environment encompasses four aspects: community nutrition environment, organizational nutrition environment, consumer nutrition environment, and information environment, which, in turn, are influenced by government policies and the food industry.

The food environment, broadly conceptualized to include any opportunity to obtain food [1], is increasingly being recognized as critical to health [5]. Further, also according to Glanz et al. [3], the food environment could be understood as a determinant of nutrition and health outcomes due to the variety of food available at different places (households, markets, restaurants, and companies) which may be poorer quality depending on the economic, social, and racial context. Although most research on the food environment has focused on assessing the community nutrition environment [6,7], the number of studies investigating the effects of the consumer nutrition environment on health outcomes has also grown [8].

The term consumer nutrition environment [3] or consumer food environment [1], used nowadays, is characterized by a set of factors that refer to foods available, such as how they are supplied or presented (their size, packaging, and portion size), the way they are placed and/or served, their nutritional quality, labeling, and nutrition claims, as well as their prices and promotions [3]. Studies have shown that price reductions [9], promotions [10], healthy food available near the entrance of the store and in cash registers [11,12], variety, tastings, and offers of free samples [13] have been positive strategies in increasing the acquisition of foods considered as healthy, such as fruits and vegetables at supermarkets.

The classification of food retailers as more or less healthy has been an important consideration in tool developed to assess the consumer food environment, such as the *Nutrition Environment Measures Survey in Stores* (NEMS-S) [14]; *Nutrition Environment Measures Survey in Restaurants* (NEMS-R) [15]; and another study in Australia [16], which considers, in the development of a scoring system, indicators such as food availability, food quality, variety, and food price.

In Brazil, assessing the healthiness of food retailers is challenging due to the diversity among retailers [17], and the lack of audit data on the consumer food environment, which would allow the inside of the stores to be checked in detail. Borges and Jaime (2019) developed and validated an audit tool for the consumer food environment–AUDITNOVA—based on the classification of foods according to the extent and purpose of their processing (NOVA classification system) [18]. This is an instrument that was innovated by using NOVA as a technical framework, since this classification also guides the recommendations of the Dietary Guidelines for the Brazilian Population. Additionally, there is growing evidence associating the consumption of ultra-processed foods, one of NOVA’s groups, with negative health outcomes, such as chronic noncommunicable diseases, overweight, and obesity [19,20].

The creation of a health index for food retailers can contribute to the advancement of research that shows the relationship among the consumer food environment, obesity, and other noncommunicable diseases, also supporting the development of public policies that regulate availability and food advertising in these spaces. AUDITNOVA allows us to audit the interior of different types of food retailers (grocery/supermarket and convenience stores, bakeries, butcheries, and others), except restaurants, fast-food restaurants, and bars, because it does not include specific variables for assessing these groups of retailers [18]. However, there is still no scoring system that classifies food retailers as more or less healthy from the data collected by AUDITNOVA in Brazil. Evidence shows that food availability, price, advertising, and placement strategies (special floor displays, end-of-aisle displays, cash register displays, island bins, and others), influence food purchases [21,22] and, therefore, should be considered when creating such indicators. Therefore, the present study aimed to develop and validate a scoring system, based on AUDITNOVA, to assess the healthiness of the consumer food environment, considering food availability, price, advertising, and placement strategies.

## 2. Materials and Methods

### 2.1. Study Type and Location

This is a methodological study that aimed to develop and validate a scoring system to assess the healthiness of the consumer food environment, considering different types of food retailers and indicators, such as availability, price, advertising, and placement strategies. The study was approved by the Research Ethics Committee of the School of Public Health under number 69045917.5.0000.5421. All those responsible for food retail were correctly informed about the research and signed the Free and Informed Consent Form.

To develop and validate the score proposed in this study, a data set from an audit process carried out inside food retailers was used. This data set was collected in the municipality of Jundiai, located in the state of Sao Paulo, Southeastern Brazil. According to the Brazilian Institute of Geography and Statistics (IBGE), Jundiai has approximately 418,962 inhabitants (in 2019) with a Human Development Index (HDI) of 0.82 (very high) and a total area of 431.207 km^2^ divided into 684 urban and rural census sectors.

### 2.2. Audit of the Consumer Food Environment

An internal audit of food retailers from 573 census tracts in the urban area of the city (representing 83.8% of the territory) was carried out. All retail food stores found on this route were audited, excluding restaurants, fast food restaurants, bars, and street markets. A total of 650 food retailers were audited and grouped into 8 categories according to the proposal of Costa et al. (2013) [17] and Machado et al. (2017) [23]: public specialized indoor fresh food markets (farmers and municipal markets); private specialized indoor fresh food markets; supermarkets; grocery stores; butchers and fishmongers, food retailers with the predominant sale of beverages; food retailers with the predominant sale of ultra-processed foods (conveniences, pharmacies, sweets and confectionery stores, and supplement stores), and bakeries.

The audit was carried out by 6 researchers (nutrition students) previously trained according to the research protocol [24]; three nutritionists worked as field supervisors. The training lasted 4 h and covered aspects such as personal presentation, filling the AUDITNOVA checklist, learning about technical specifications of food, learning the food classification system according to NOVA proposed by Monteiro et al. [25], and presentation of the Informed Consent Form. The auditing process lasted 4 months, starting in December 2017 and ending in April 2018.

The audit was performed using the AUDITNOVA tool, which allows the collection of availability and price information for 66 types of food (selected using food acquisition data in Brazil from the Household Expenditure Survey—2008/2009), of which 35 were fresh and minimally processed foods, 6 were culinary ingredients, 7 were processed foods, and 18 were ultra-processed foods [18]. Information on different advertising claims was also collected both for fresh and minimally processed and ultra-processed foods. Among the types of advertising claim, we can list health and well-being, practicality, distribution of free samples, highlight taste, color and texture, nutritional and functional properties, among others that were measured at strategic points (near the store entrance, near the cash register, end-of-aisle, island bins, and special floor displays). It should be noted that AUDITNOVA is just a tool to collect data and does not have a scoring system to classify food retailers as healthy. In this sense, to be able to generate the score proposed in this study, data must be collected by the AUDITNOVA tool.

### 2.3. Development of the Consumer Food Environment Healthiness Score (CFEHS)

To elaborate the Consumer Food Environment Healthiness Score (CFEHS), the variables collected using the AUDITNOVA served as the basis for creating a set of indicators classified into two dimensions: 1. food dimension, composed of the indicator availability and promotional price, and 2. environmental dimension, composed of the indicators advertising/information and placement. Methodologically, the choice of these specific indicators and dimensions occurred as systematic reviews highlight availability, price, advertising, as well placement strategies as the key determinants of food purchases in the consumer food environment [7,26].

In each dimension, a set of indicators composed of a subset of dichotomous variables (yes or no) selected from AUDITNOVA was created; Table 1 shows more details. A score interval was attributed to each indicator, depending on their healthiness in the food consumer’s environment. These healthiness scores were based on the recommendations of the Dietary Guidelines for the Brazilian Population [27]. According to the guidelines, foods from NOVA’s first three groups—fresh or minimally processed foods, culinary ingredients, and processed foods—can compose healthy eating patterns, since they are part of culinary preparations and balanced meals, thus receiving a positive score in this study. The Brazilian Guidelines also recommend prioritizing vegetable origin foods rather than animal origin foods. For ultra-processed foods (Group 4), the recommendation is to avoid them, which is why they received a negative score.

In the food dimension, the total point on each indicator considers food availability and, in some cases, if the retailer offers promotional prices. This factor was accounted only for foods that are markers of healthy eating (fruits, vegetables, beans, and fish) or unhealthy eating (soft drinks, nectar, and sweets) according to Brazilian national studies [30].

For example, in the food dimension, the indicator “Fruits” is composed of a variable set on the availability of different types of fruits (orange, banana, papaya, apple, watermelon, and other fruits) collected by AUDITNOVA at the food retailer. In the case of the availability of 1 or 2 fruits, three points should be accounted for: for 3 to 5 fruits, 6 points; for 6 fruits, 9 points. Since fruits are markers of healthy eating, if there are at least 3 items with a promotional price, 3 extra points should be accounted for. Opposite points are attributed to the indicator “Ultra-processed foods”, considering the total counting of items collected by AUDITNOVA (Table 1).

For the environment dimension, points were awarded according to the scoring parameter related to the Dietary Guidelines; that is, if advertising and placement strategies in the consumer food environment were related to the group of fresh or minimally processed foods, the score was positive; if it was related to the group of ultra-processed foods, it was negative. The full list of variables used to compose the Consumer Food Environment Healthiness Score (CFEHS) in the food and environment dimensions can be seen in Table 1.

### 2.4. Statistical Analysis

The scores for the food and environment dimensions were computed using the simple sum of the indicators for each dimension, standardized for the scale from 0 to 100 points. The higher the score is (closer to 100), the healthier the food retailers are. To obtain the total CFEHS, the average between the scores of the two dimensions was calculated, so that they both had the same weight in the final score. Descriptive analysis was performed, and the distribution of variables was verified by the Kolmogorov–Smirnov test. The total CFEHS variables, food score, and environment score were described by the median and interquartile range (P25–P75). To analyze the statistical measures of the data set, such as variability, mean, and outliers, Box-Plot graphs of the total CFEHS variables, food dimension score, and environment dimension score according to types of food retailers were constructed.

To compare the median of CFEHS and its dimensions between types of food retailers, the Kruskal–Wallis test for nonparametric variables was used.

The construct validity of the CFEHS was evaluated to identify whether the score reflected the desired theoretical concept, in this case, the healthiness of the consumer food environment [31]. For this, it was analyzed if the score could classify three food retailer groups previously proposed to identify food deserts in Brazil [32], which were adapted for this study: Group 1. food retailers with the predominant sale of fresh or minimally processed foods (composed of public specialized indoor fresh food markets; private specialized indoor fresh food markets, and butchers and fishmongers); Group 2. mixed food retailers (composed of bakeries, supermarkets, and grocery stores); and Group 3. food retailers with the predominant sale of ultra-processed foods (composed of conveniences, pharmacies, sweets and confectionery stores, supplement stores, and food retailers with the predominant sale of beverages). The scores of the three categories in the CFEHS and its dimensions were compared using the Kruskal–Wallis test and the Dunn test. To confirm the validity of the CFEHS and its dimensions, it was expected that they could discriminate between the three categories of establishment, so that category 1 retailers had higher scores than those in category 2, and that both categories had higher scores than those in category 3.

Cronbach’s alpha (α) coefficients were calculated to determine the internal consistency of the total CFEHS and its comprising dimensions. Cronbach’s α values > 0.70 indicate good internal data consistency [33].

All analyses were conducted in the STATA 15 statistical package. Statistically significant differences were considered to be *p* < 0.01.

## 3. Results

Of the 650 commercial retailers studied, 39.9% were food retailers with the predominant sale of ultra-processed foods and 25.2% were grocery stores, which together accounted for over 65% of the retailers selling food in the region; of the remaining 35.0%, around 14.0% were bakeries, 6.0% were butchers and fishmongers, 6.0% were private and public specialized indoor fresh food markets, 5.0% were supermarkets, and 4.0% were food retailers with the predominant sale of beverages (Table 2).

The CFEHS presented a median value of 33.7 (p25 = 26.9 and p75 = 42.1). The food retailers with the highest CFEHS medians were public and private specialized indoor fresh food markets, and those with the lowest medians were bakeries and food retailers with the predominant sale of ultra-processed of foods. When analyzing the food dimension score, public specialized indoor fresh food markets have a higher score, followed by private fresh food markets and supermarkets. For the environment dimension, public and private specialized indoor fresh food markets also have higher scores (Table 2).

**Figure 1:**
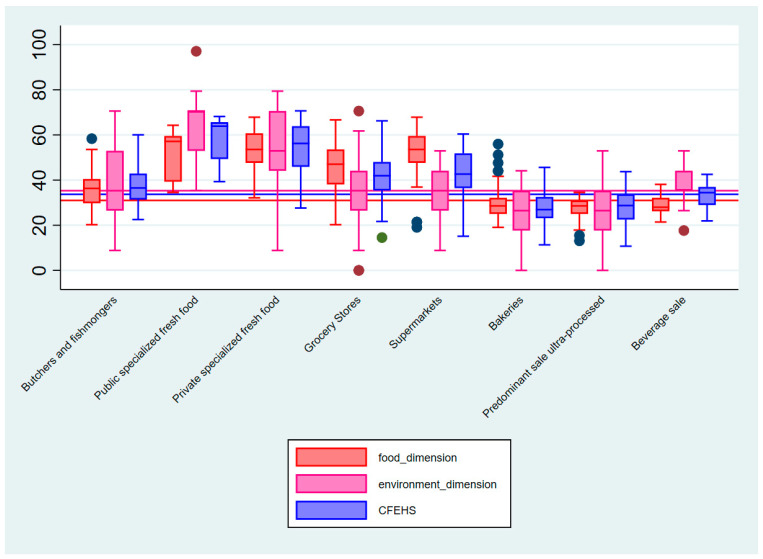
Consumer Food Environment Healthiness Score Box Plot and its food and environment dimensions according to different categories of food retailers. Brazil.

Figure 1 illustrates the Box Plot with the extent and variability of data according to the different types of food retailers. It is possible to visualize in the box the median, minimum and maximum values, and interquartile ranges for the scores. It is also possible to verify variations in the CFEHS score and its dimensions within the same category of establishments and between different types. The three continuous straight lines in the x-axis show the mean score in their three dimensions. In the food dimension, we observed outliers (extreme values) for both butchers and fishmongers as well as for bakeries and food retailers with the predominant sale of ultra-processed food; still, in this dimension, we also observed that the public specialized indoor fresh food markets have the highest median. In the environment dimension, we observed outlier values into two categories as public specialized indoor fresh food markets (also with this higher median size) and grocery stores. In CFEHS outliers, we found that grocery stores and the public specialized fresh-food market showed the high median.

In the construct validation analysis, it was observed that CFEHS could satisfactorily classify the extreme groups 1 (predominance of fresh and minimally processed foods) and 3 (predominance of ultra-processed foods) and groups 2 (mixed food retailers) and 3, but not groups 1 and 2. For the food dimension, the same associations as the total CFEHS were identified; however, the environment dimension adequately classified all groups (Table 3).

The results of Cronbach’s alpha showed excellent internal consistency for the total CFEHS (α = 0.91) and for the food dimension (α = 0.91), whereas the values for the environment dimension (α = 0.60) were lower.

## 4. Discussion

This study developed, validated, and tested a Consumer Food Environment Healthiness Score (CFEHS) composed of two dimensions, one related to the availability and promotional price, named the food dimension, and the other related to advertising and placement strategies, named the environment dimension. In the construction of these indicators, high internal consistency of the data was observed, except for the environmental dimension. CFEHS showed the ability to classify food retailers that were previously reported in the literature to map food deserts in Brazil [32]. Additionally, when creating this score, the key determinants of consumer food choices in food retailers were considered, the availability of food [11,12] and advertising [14] coupled placement strategies [34]. This was the first Brazilian study to consider the degree of food processing as a theoretical basis for the development of a healthiness score for the consumer food environment.

Studies that assess the healthiness of the consumer food environment consider as healthy the food retailers that presents a set of factors considered empowering for healthy food choices, such as offering a greater variety of fresh foods; having a fruit and vegetable section at the entrance of the store and or in a special floor display; carrying out healthy food price promotions; and, at the same time, presenting an internal environment with a lower number of advertisements [14,16,35,36,37,38]. In this study, we chose to study two dimensions in addition to the total score because of the complexity of the determinants of food choices in food retailers. In this sense, a retailer may present a high availability of healthy foods, but a massive presence of advertising and many placement strategies to promote unhealthy food products, making the retailer less likely to promote good nutrition and health. For a food retailer to be considered healthy using the scores proposed in this study, its scores should be close to 100 for both the food dimension and the environment dimension, since both are related to the consumer’s food choices in these spaces.

In the international scenario, the study proposed by Glanz et al. (2007) [14], named the *Nutrition Environment Measures Survey in Stores* (NEMS-S), developed a health score for food retailers ranging from 0 to 50 points. In the construction of this score, information on availability, price, and quality of the food offered was used. Placement strategies and advertising are left out in the NEMS-S, and the scoring method adopted was also developed for an international context, with the food pyramid as its theoretical framework for healthy eating [14]. The NEMS-S health score considers healthy foods as those with a reduction in some macronutrients (example: lean meat and low-fat dairy) or whole versions (example: whole grain bread) and does not consider the degree of food processing.

In the study of the Obesogenic Environment of Sao Paulo (ESAO-SP), carried out by Duran et al. (2015) [38], an instrument for assessing the consumer food environment was developed and validated, and a scale to measure the availability of healthy foods in food retailers, named *Healthy Food Store Index* (HFSI), was proposed. This index comprises different indicators of the consumer food environment, and it varies from 1 to 16; the higher the value is, the better the availability of healthy foods is, and, consequently, the lower the availability of ultra-processed foods is. This indicator also assesses the advertising of ultra-processed foods and the presence of vegetables near the entrance of the store, items also considered in the construction of CFEHS. The main differences between CFEHS and HFSI are in the incorporations of the new food group markers of a healthy diet as culinary ingredients and processed foods, in addition to the traditional indicators of fruits and vegetables and the inclusion of promotional prices and placement strategies, making CFEHS more complete and in consonance with national recommendations for healthy eating.

In Brazil, two important studies using data from the Household Expenditure Survey carried out by IBGE found that the primary place for food acquisition among Brazilians is the supermarket [17,23]. When evaluating the performance of the supermarket chain category among the scores proposed in this study, it was possible to observe a high score for the food dimension, but low for the environment dimension. By grouping all supermarkets, grocery stores, and bakeries into a single group of mixed retailers, it was possible to observe a lower score for the environment dimension compared to the group of food retailers with the predominant sale of fresh or minimally processed foods, although no significant differences were observed for the food dimension and for the total CFEHS. This indicates that these types of food retailers offer a wide range of foods that make up healthy eating patterns, but at the same time, their environmental (advertising and placement strategies) characteristics tend to favor the purchase of unhealthy foods.

This study analyzed eight different categories of food retailers present in a municipality of Brazil, and most of them were classified by the predominant sale of ultra-processed foods (comprising convenience stores, candy stores, cake shops, ice cream shops, and pharmacies), presenting the worst performance in the healthiness score. Butchers and fishmongers and public and private specialized indoor fresh food markets had a higher CFEHS median and can be considered as the best food retailers to help people achieve the recommendations of the Dietary Guidelines for the Brazilian Population [27], as they offer availability and good prices for healthy food and, at the same time, a low prevalence of advertising and placement strategies that promote ultra-processed foods.

It is challenging to compare different categories of retailers and their healthiness in different countries since their characteristics, as well as the classification rules of food retailers and the indicators and audit tools available for their evaluation, do not always follow the same criteria and standardization [26]. In the United States of America, small retail stores (named grocery stores or small retailers), which could be equivalent to our grocery stores, are associated with a better availability of healthy foods than those named corner stores (equivalent to our convenience stores) [39].

Although the data used in this study are from a medium-sized municipality, we believe that they could be used to assess the healthiness of food retailers in other regions of Brazil because, as seen, the score was able to classify groups of food retailers, created by the Brazilian government, and used to identify food deserts in all Brazilian territory [32]. It also has the advantage of being a more complete score that encompasses not only dimensions of food availability but also price, advertising, and placement strategies. As we know that almost half of the Brazilian population makes food purchases in supermarkets [21], the score produced in this study could be useful to assess the healthiness of these locations. In Brazil, there is still no consensus; the supermarket, in one study, was a promoter of healthy foods purchases, such as fruits and vegetables [40], and in another, was a promoter of ultra-processed foods purchases [21,23].

It is worth highlighting some strengths of this study. This is an in-depth analysis of the internal environment of different categories of food retailers located in a medium-sized municipality in Brazil. The use of the AUDITNOVA tool made it possible to collect, at the consumer food environment level, information on availability, price, advertising, and placement strategies not only for fruit and vegetables but also for the other NOVA food groups. The developed score may support the territorial identification of healthy food environments in which individuals and communities can exercise healthy behavior. However, the study also has some limitations, such as not including other food retailers, such as restaurants and bars, in the audit performed because the AUDITNOVA tool did not allow it. It is known that each year the Brazilian population consumes more food out of the home [41] and that it is also necessary to expand studies on the health of these environments and how they can influence the food purchasing in Brazil. The quality indicator generally used to evaluate fruits and vegetables was not used in this study in the construction of the score because, according to the theoretical framework adopted, a quality meal involves the presence of different food groups.

According to validity and reliability results, CFEHS proved to be an adequate tool for classifying and identifying food retailers according to their healthiness. The low value of Cronbach’s alpha for the environment dimension may be related to the fact that it has a smaller number of indicators, since the alpha coefficient is sensitive to the number of items [42]. However, this factor does not represent a limitation of the score, since healthiness is understood here as a construct formed by the inseparable combination of environmental characteristics and the set of foods being sold. Therefore, the use of these indicators is more appropriate as components of a broader concept of healthiness and not as isolated indicators.

The present study proposed a score to assess the consumer food environment, comprising food and environment dimensions, to provide a general assessment of the healthiness of food retailers, showing that public specialized indoor fresh food markets present the healthiest score. The applied CFEHS showed the ability to discriminate groups of retailers reported as healthy and unhealthy according to the degree of processing of the commercialized foods. Every aspect measured in this study may be useful in future research that aims to measure the relationship between internal aspects of the consumer food environment and its relationship with the quality of the diet, as well as assessing the effects of interventions on the consumer food environment with multiple components.

## Figures and Tables

**Table 1:** Indicators comprising the food and environment dimensions of the Consumer Food Environment Healthiness Score and total scores assigned according to NOVA food classification groups.

Food Dimension (Availability and Promotional Price)				
NOVA Group	CFEHS Indicators (in bold)	SCORE		Indicator Total Score
	AUDITNOVA Variables (*in italic*)	Availability	Promotional Price	
Vegetable origin, fresh or minimally processed (base = 3 points)	**Fruits**			12
	*Orange; banana; papaya; apple; watermelon; other fruits (yes or no)*	1 to 2 items = 3 3 to 5 items = 6 6 items = 9	> 2 promotional items = 3	
	**Vegetables**			12
	*Tomato (for salad); onion; crisp lettuce; carrots; Brazilian zucchini; chayote; parsley and chives; other vegetables (yes or no)*	1 to 3 items = 3 4 to 7 items = 6 8 items = 9	> 2 promotional items = 3	
	**Roots, tubers, and corn**			9
	*Potatoes; cassava; other roots and tubers; corn on the cob (yes or no)*	1 item = 3 2 to 3 items = 6 4 items = 9		
	**Beans and rice**			
	*Kidney beans; black beans; white rice (yes or no)*	Rice only = 3 Beans only = 3 Rice and beans = 6	At least 1 bean with promotional price = 3	
Animal origin, fresh or minimally processed (base = 2 points)	**Meat and eggs**			8
	*Large white chicken egg; other types of eggs; prime beef (flank); beef (neck); whole chicken (with bone and skin); chicken breast; fish (any species) (yes or no)*	Beef only = 2 Chicken or eggs (at least 1 type; regardless of beef) = 4 Fish (regardless of other meats) = 6	Fish with promotional price = 2	
	**Milk**			2
	*UHT ^a^ whole cow’s milk*	Whole cow’s milk = 2		
Culinary Ingredients (base = 1 point)	**Culinary Ingredients**			
	*Salted Butter; soy oil; extra virgin olive oil; refined salt; coarse sugar; white or refined table sugar (yes or no)*	1 item = 1 2 to 4 items = 2 5 to 6 items = 3		3
Processed (base = 1 point)	**Processed bread**			1
	*Freshly baked Bread (yes or no)*	Bread = 1		
Ultra-processed (base = −3 points)	**Ultra-processed foods and beverages**			
	*Hot dog sausage; pork sausage; fermented milk drink strawberry flavor; instant noodles (chicken flavor); powdered seasoning; sliced bread; breakfast cereals (corn flakes); pizza ready to heat; ice cream; regular soft drinks (350mL can and 2L); zero, light or diet soft drinks; nectar in Tetra Pak box; powdered soft drink; corn snack; chocolate filled cookie; milk chocolate; candies (yes or no)*	−1 for each available item	At least one soft drink with promotional price = −3	−27
			Soft drink or nectar with promotional price = −3	
			At least one treat with a promotional price = −3	
**Total Food Dimension Variation**				**−27 a 56**
**Environment dimension (advertising and placement strategies)**				
**NOVA Group**	**CFEHS indicators (in bold)**		**SCORE**	**Indicator total score**
	**AUDITNOVA variables (*in italic*)**			
Fresh or minimally processed foods (base = 3 points)	**Presence of fruit and vegetables at the store entrance**			
	*Is the fruit and vegetable section located near the main entrance in the store?^b^ (yes or no)*		Yes = 3	3
	**Advertisement of fresh/minimally processed foods at the store entrance**			
	*Is there advertisement of fresh/minimally processed foods at the store entrance? (yes or no)*		Yes = 3	3
	**Advertisement of fresh/minimally processed foods at the store outside**			
	*Is there advertisement of fresh/minimally processed foods at the store outside? (yes or no)*		Yes = 3	3
	**Advertisement of fresh/minimally processed foods inside the store**			9
	*Flags; posters/banners; displays; tabloids with information about prices or promotions; folders or leaflets with recipes and culinary tips; or the following types of advertisements: showing some functional property; associating with physical activity; highlighting health and well-being; appealing practicality; “3 for the price of 2” type; highlighting sensorial proprieties; highlighting new launches; offering free gifts (tie-in sale) (yes or no)*		1 type = 3 2 to 3 types = 6 4 or more types = 9	
Ultra-processed (base = −3 points)	**Advertisement of ultra-processed foods at the store entrance**		Yes = −3 points	−3
	*Is there advertisement of ultra-processed foods at the store entrance? (yes or no)*			
	**Advertisement of ultra-processed foods at the store outside**		Yes = −3 points	−3
	*Is there advertisement of ultra-processed foods at the store outside? (yes or no)*			
	**Presence of ultra-processed foods at the store check-out (cash register)?**		Yes = −3 points	−3
	*Are there ultra-processed foods available at the store check-outs (cash register)?*			
	**Presence of advertisement of ultra-processed foods inside the store**			−9
	*Food tasting counter; free sample distribution; displays; island bins; end-of-aisle; or the following types of advertisements: claiming health and well-being; appealing practicality; claiming functional properties; “3 for the price of 2”; emphasizing sensorial proprieties; highlighting new launches; offering free gifts (tie-in sale) (yes or no)*		1 a 3 types = −3 4 a 5 types = −6 6 or more types= −9	
**Total Environment Dimension Variation**				**−18 to 15**
**Total Cfehs Variation**				**−46 to 71**

^a^ Ultra-high-temperature (UHT) pasteurization involves heating milk or cream from 138 to 150 °C (280 to 302 °F) for one or two seconds; ^b^ could be understood as a display of fruits or vegetables near their cash registers [28], or strategic placement of healthy foods at supermarket [29]. NOVA = NOVA food classification proposed by Monteiro et al. [25] which classify foods in four groups: 1. unprocessed or minimally processed foods, 2. Culinary ingredients, 3. Processed Foods and, 4. Ultra-processed Foods.

**Table 2:** Consumer Food Environment Healthiness Score median (p25 and p75) and its food and environment dimensions according to different categories of commercial retailers in Brazil.

Food Retailers	Total	Percent	Food Dimension Score		Environment Dimension Score				CFEHS (Environment and Food)		
	N	%	Median *	P25	p75	Median *	P25	P75	Median *	P25	P75
Public specialized indoor fresh food markets	15	2.3	57.1	39.3	59.5	70.6	52.9	70.6	63.9	49.3	65.7
Private specialized indoor fresh food markets	23	3.5	53.6	47.6	60.7	52.9	44.1	70.6	56.2	45.9	63.9
Supermarket	31	4.8	53.6	47.6	59.5	35.3	26.5	44.1	42.7	36.5	51.8
Grocery Stores	164	25.2	47.0	38.1	53.6	35.3	26.5	44.1	41.9	35.4	48.0
Butchers and fishmongers	38	5.9	36.3	29.8	40.5	35.3	26.5	52.9	36.5	31.3	42.9
Food retailers with predominant sale of beverages	26	4.0	28.0	26.2	32.1	35.3	35.3	44.1	34.4	29.0	36.9
Food retailers with predominant sale of ultra-processed foods **	259	39.9	28.6	25.0	31.0	26.5	17.7	35.3	28.7	22.5	33.7
Bakeries	94	14.5	28.6	25.0	32.1	26.5	17.7	35.3	26.9	23.1	32.5
**TOTAL**	650	100.0	31.0	27.4	44.1	35.3	26.5	44.1	33.7	26.9	42.1

CFEHS: Consumer Food Environment Healthiness Score; * *p*-value < 0.0001 using the Kruskal–Wallis method, showing a significant difference in all scores between types of retailers; ** conveniences, pharmacies, sweets and confectionery stores, and supplement stores.

**Table 3:** Mean (SD) and median (p25 and p75) of the Consumer Food Environment Healthiness Score and its food and environment dimensions according to three groups of commercial retailers in Brazil.

Total Score and Dimensions	Group 1 (*n* = 76)		Group 2 (*n* = 289)		Group 3 (*n* = 285)			Group1 vs. Group2	Group1 vs. Group3	Group2 vs. Group3
	Mean (SD)	Median	Mean (SD)	Median	Mean (SD)	Median	*p*-Value *	*p*-Value	*p*-Value	*p*-Value
Food Dimension Score	44.0 (12.3)	42.3	41.4 (12.4)	40.5	28.0 (3.7)	28.6	0.000 *	0.081	0.000 **	0.000 **
Environment Dimension Score	49.7 (18.6)	52.9	32.4 (13.3)	35.3	29.7 (13.7)	35.3	0.000 *	0.000 **	0.000 **	0.000 **
Total Score (Environment and Food)—CFEHS	46.8 (13.4)	44.7	36.9 (10.7)	36.7	28.8 (7.9)	28.9	0.000 *	0.292	0.000 **	0.000 **

Group 1. food retailers with predominant sale of fresh or minimally processed foods (public specialized indoor fresh food markets; private specialized indoor fresh food markets; butchers and fishmongers); Group 2. mixed food retailers (bakeries, supermarkets, grocery stores); Group 3. food retailers with predominant sale of ultra-processed foods (conveniences, pharmacies, sweets and confectionery stores, supplement stores and food retailers with predominant sale of beverages); * Kruskal–Wallis test for nonparametric variables, ** Dunn test; SD: standard deviation; p25: 25th percentile; p75: 75th percentile. CFEHS: Consumer Food Environment Healthiness Score.

## Data Availability

The data presented in this study are available on request from the corresponding author. The data are not publicly available due to these data belonging exclusively to the research group of the Faculty of Public Health—USP.
